# Jacobian integration method increases the statistical power to measure gray matter atrophy in multiple sclerosis^☆^

**DOI:** 10.1016/j.nicl.2013.10.015

**Published:** 2013-10-29

**Authors:** Kunio Nakamura, Nicolas Guizard, Vladimir S. Fonov, Sridar Narayanan, D. Louis Collins, Douglas L. Arnold

**Affiliations:** a McConnell Brain Imaging Centre, Montreal Neurological Institute, McGill University, 3801 University Street, Montreal, Quebec H3A 2B4, Canada; b NeuroRx Research, 3575 Park Avenue, Suite #5322, Montreal, Quebec H2X 4B3, Canada

**Keywords:** Multiple sclerosis, Magnetic resonance imaging, Gray matter, Atrophy, Sample size, Jacobian integration

## Abstract

Gray matter atrophy provides important insights into neurodegeneration in multiple sclerosis (MS) and can be used as a marker of neuroprotection in clinical trials. Jacobian integration is a method for measuring volume change that uses integration of the local Jacobian determinants of the nonlinear deformation field registering two images, and is a promising tool for measuring gray matter atrophy. Our main objective was to compare the statistical power of the Jacobian integration method to commonly used methods in terms of the sample size required to detect a treatment effect on gray matter atrophy. We used multi-center longitudinal data from relapsing–remitting MS patients and evaluated combinations of cross-sectional and longitudinal pre-processing with SIENAX/FSL, SPM, and FreeSurfer, as well as the Jacobian integration method. The Jacobian integration method outperformed these other commonly used methods, reducing the required sample size by a factor of 4–5. The results demonstrate the advantage of using the Jacobian integration method to assess neuroprotection in MS clinical trials.

## Highlights

• We compared sample sizes to detect a treatment effect on gray matter atrophy.
• Jacobian integration method outperformed SIENAX, SPM, and FreeSurfer.
• Sample sizes were reduced by 4 to 5 fold with Jacobian integration method.
• Use of Jacobian integration may be advantageous for assessing neuroprotection.

## 1 Introduction

Multiple sclerosis (MS) is an inflammatory, demyelinating disease of the central nervous system. Although multiple focal lesions in white matter are the pathologic and imaging hallmarks of MS, gray matter is also involved. Gray matter pathology, which has been known from early post-mortem studies (Dawson, 1916) but overlooked for many decades, has recently become a new focus of MS research (Kutzelnigg et al., 2005; Lucchinetti et al., 2011). Several postmortem (Bo et al., 2007; Kutzelnigg et al., 2005), in vivo magnetic resonance imaging (MRI) (Mainero et al., 2009), and MR spectroscopy studies (Caramanos et al., 2009) have shown that gray matter pathology appears to be independent of white matter pathology, suggesting distinct mechanisms of tissue destruction. Pathological studies have shown that there is significant gray matter demyelination in MS, the extent of which can exceed that of white matter (Geurts et al., 2012; Kutzelnigg et al., 2005). However, cortical lesions are rarely visible on conventional MRI (Geurts et al., 2005a, 2008). Advanced MRI techniques such as double inversion recovery and phase-sensitive inversion recovery can improve sensitivity to leukocortical and intracortical lesions (Geurts et al., 2005b; Nelson et al., 2007) but fail to capture the large bands of subpial demyelination seen on histopathology (Seewann et al., 2012). Tissue loss in gray matter (gray matter atrophy), which apparently results from lesional as well as non-lesional pathology (Wegner et al., 2006) and represents overall destructive pathology including neurodegeneration, can be measured by conventional MRI.

Measures of cortical gray matter tissue loss or atrophy are clinically relevant, as they correlate with cognitive impairment (Amato et al., 2007), are more closely associated with physical disability than whole brain atrophy (Fisher et al., 2008), and appear to be less influenced by so-called “pseudoatrophy” than whole brain or white matter atrophy (Nakamura et al., 2010; Tiberio et al., 2005). Indeed, these properties make cortical gray matter atrophy attractive as an outcome measure in clinical trials, particularly as therapeutic targets shift from suppression of inflammation to neuroprotection and remyelination.

The longitudinal measurement of cortical volume change on MRI is not an easy task because the cortex is thin and convoluted, and the relaxation behavior of both cortex and white matter can change with pathology. To be useful as an outcome measure in MS, it is critical to determine an optimal strategy to quantify gray matter atrophy with high statistical power. The objectives of this study were: (1) to assess the reproducibility of various analysis pipelines to measure cortical or gray matter volume, (2) to quantify cortical or gray matter atrophy over time in an MS population, (3) to compare these pipelines in terms of required sample size, and (4) to assess factors in study design (image resolution and study duration) that influence the statistical power to detect a clinical effect on the rate of cortical atrophy over time.

## 2 Material and methods

We used a scan–rescan dataset to calculate reproducibility and a longitudinal clinical study of MS patients to measure the required sample sizes to detect gray matter atrophy.

### 2.1 Subjects

Subjects for the scan–rescan dataset were 20 healthy normal controls (age = 30 ± 4 years, 10 females) (Aubert-Broche et al., 2006). Subjects for the longitudinal dataset came from a multi-center clinical study (Assessment Study of Steroid Effect in Relapsing Multiple Sclerosis Subjects Treated with Glatiramer Acetate, ASSERT, NCT00203047) involving 414 relapsing–remitting MS (RRMS) patients. A cohort of 287 patients (mean baseline age = 39.9 ± 9.0, proportion of female = 73.2%) who completed at least two MRI sessions was studied here. All patients were randomized to either with glatiramer acetate alone or with glatiramer acetate plus 1250 mg of prednisone given orally for 5 days every 4 months.

### 2.2 Imaging

The scan–rescan MRIs were previously obtained T1-weighted 3D spoiled gradient-recalled echo images [echo time (TE) = 9.2 ms, repetition time (TR) = 22 ms, flip angle (FA) = 30°, resolution = 1.0 × 1.0 × 1.0 mm^3^]. The images were acquired twice on the same day from 1.5 Tesla Siemens Sonata Vision scanner.

The longitudinal data were acquired at 63 different clinical sites using 1.0 T (n = 2), 1.5 T (n = 57), or 3.0 T (n = 4) scanners. The manufacturers included Philips (n = 15), Siemens (n = 18), General Electric (n = 25), and Marconi (n = 5). Relevant MRI sequences included: (a) axial proton density (PD)-weighted spin echo [TE = 10–17 ms, TR = 2000–3800 ms, in-plane resolution = 0.977 × 0.977 mm^2^, slice thickness = 3 mm], (b) axial T2-weighted spin echo images [TE = 77–96 ms, TR = 3267–7767 ms, in-plane resolution = 0.977 × 0.977 mm^2^, slice thickness = 3 mm], (c) sagittal high-resolution 3D T1-weighted gradient echo image [TE = 4–10 ms, TR = 15–24 ms, FA = 30°, resolution = 1.5 × 1.0 × 1.0 mm^3^], and (d) axial standard-resolution 3D T1-weighted gradient echo image [TE = 5–11 ms, TR = 28–34 ms, FA = 30°, resolution = 1.0 × 1.0 × 3.0 mm^3^]. Subjects were scanned annually for up to 3 years.

### 2.3 Segmentation of MS lesions

T2-lesions in white matter were automatically segmented using a multispectral Bayesian classifier (Francis, 2004) with PD-weighted, T2-weighted, and T1-weighted images, and then reviewed by experts and manually corrected as necessary. No cortical gray matter lesions were identified, as the scanning sequence was not designed to be sensitive to gray matter lesions.

### 2.4 Image analysis

The T1-weighted images for each subject were analyzed by combinations of cross-sectional and longitudinal pre-processing with cross-sectional segmentation-based and longitudinal registration-based algorithms. The following section describes the details of the pre-processing and atrophy measurement methods. All methods were fully-automated except for the MS-lesion segmentation described above.

**Fig. 1:**
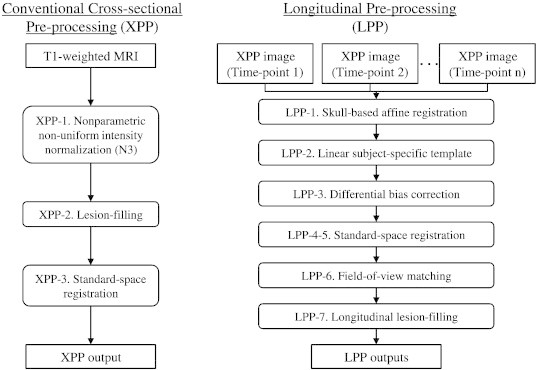
Flowchart describing the cross-sectional (XPP) and longitudinal (LPP) pre-processing pipelines.

Conventional cross-sectional pre-processing (XPP): As shown in Fig. 1, XPP consisted of (XPP-1) N3 intensity-non-uniformity correction (Sled et al., 1998); (XPP-2) MS-lesion filling (Battaglini et al., 2012) (to reduce bias in gray matter volumes due to the impact of variable white matter MS lesion loads on image intensity distributions) (Nakamura and Fisher, 2009); and (XPP-3) standard ICBM-space registration (using the ICBM 2009c Nonlinear Symmetric Template) (Fonov et al., 2009), using a hierarchical registration technique (Nakamura, 2011). Briefly, the hierarchical registration procedure involved estimating the affine transformation parameters in multiple steps: (1) two rotations (y- and z-rotations) by maximizing the left and right inter-hemispheric symmetry, (2) x-rotation and z-translation by normalized mutual information (NMI) registration to align anterior–posterior on the y-axis, (3) multi-seed optimization for a global scaling factor using NMI, and (4) three scaling and three shearing parameters again estimated with NMI. The Nelder–Mead simplex method was used to optimize each step with NMI as the cost function (Pluim et al., 2003). For lesion filling, the mean and standard deviation of normal-appearing white matter (NAWM) were estimated from initial segmentation of normal-appearing brain tissue using FMRIB's Automated Segmentation Tool (FAST) (Zhang et al., 2001).

Longitudinal pre-processing (LPP): The longitudinal pre-processing began with the XPP and added the following: (LPP-1) Intra-subject registration using *pairreg* from FSL, which includes skull-based corrections for scaling and skewing between each pair of images (Jenkinson et al., 2002), that corrects for potential voxel size mis-calibration and, to some extent, geometric distortion (Caramanos et al., 2010); (LPP-2) An unbiased subject-specific linear template using all combinations (between each time-point) of linear transformations (Nakamura et al., 2011). The matrix average was calculated as in Leung et al. (2012) using octave (http://www.octave.org); (LPP-3) Differential intensity correction, which estimates the bias field as a median-filtered ratio map with respect to the subject-specific template (Leung et al., 2012; Lewis and Fox, 2004); (LPP-4) Template-to-standard-space registration using the hierarchical method, as previously described; (LPP-5) Consistent standard space (ICBM 2009c Nonlinear Symmetric Template (Fonov et al., 2009)) registration by concatenating the native-to-template affine registration matrix and template-to-standard-space registration matrix; (LPP-6) Field-of-view matching by removing all voxels that were not in the image at any time-point, and (LPP-7) Longitudinal lesion-filling using combined lesion masks, where the lesion masks from each time-point were transformed to the subject-specific template, combined, transformed back to the native space, and filled with NAWM intensities similar to the lesion filling in XPP (Nakamura et al., 2010). The flowchart of the two pre-processing pipelines is shown in Fig. 1.

Statistical Parametric Mapping (SPM, http://www.fil.ion.ucl.ac.uk/spm) is a software suite of MATLAB functions and subroutines. We used the latest version, SPM8b. Of the many pipelines in SPM8b, we are interested in the “Segment” function (Ashburner and Friston, 2005). It is a *cross*-*sectional algorithm* where each image is independently analyzed. This segmentation produces tissue probability maps from which maps of gray matter, white matter, and cerebrospinal fluid classes are obtained. The tissue class with the highest probability is assigned at that voxel. For this work, we are interested only in the gray matter voxels. Since SPM requires good initial spatial normalization, we performed linear spatial normalization prior to SPM analysis using the hierarchical registration method in XPP (Nakamura, 2011) with the stereotactic ICBM 2009c Nonlinear Symmetric Template image (Fonov et al., 2009). The resulting volume, therefore, is a head-size normalized *total* (= cortical + deep + cerebellar) gray matter volume.

SIENAX is the *cross*-*sectional version* of the Structural Image Evaluation using Normalization of Atrophy (SIENA) method (Smith et al., 2002) and is part of FSL (http://www.fmrib.ox.ac.uk/fsl/). Currently, SIENA cannot measure cortical atrophy and was not used in this study. Briefly, in SIENAX, the brain is extracted from the volume using the Brain Extraction Tool (BET) (Smith, 2002) and then classified using FAST into gray matter, white matter, and cerebrospinal fluid (Zhang et al., 2001). FAST corrects for spatial intensity variations as well as partial volume, and uses a hidden Markov random field model and expectation–maximization algorithm (Zhang et al., 2001). SIENAX calculates a v-scaling factor to normalize the brain volumes so that they are comparable in the standard stereotaxic space. This scaling is the determinant of the skull-constrained brain registration matrix that registers the subject MRI and standard template. In the end, SIENAX outputs normalized and non-normalized volumes for cortical and total gray matter. In this study, we used normalized cortical volume.

FreeSurfer is a freely available image analysis package (http://surfer.nmr.mgh.harvard.edu) that has both cortical surface reconstruction (Dale et al., 1999) and volumetric segmentation (Fischl et al., 2002, 2004a,b). In this study, the surface-based cortical thickness is used to measure cortical gray matter atrophy. The images are analyzed first cross-sectionally; then the unbiased longitudinal scheme is applied to improve the consistency (Reuter et al., 2012). XPP or LPP is not applied for FreeSurfer because FreeSurfer has its own longitudinal pipeline. The FreeSurfer version is 5.1.

The Jacobian integration method is a longitudinal registration-based method and a type of tensor-based morphometry (Ashburner et al., 1998). We used a variant of the longitudinal pipeline developed in the Image Processing Laboratory at the McConnell Brain Imaging Centre at the Montreal Neurological Institute (Guizard et al., 2012). Briefly, the Jacobian integration method consisted of the following: (1) skull-based intra-subject registration using *pairreg* (Jenkinson et al., 2002), (2) transformation and resampling of both images into an isotropic halfway space using sinc interpolation, (3) symmetric nonlinear registration of the two affine-halfway-transformed images using SyN (Avants et al., 2008), (4) calculation of the local Jacobian determinants of nonlinear displacement fields, and (5) integration of Jacobian determinants within the baseline cortical masks obtained from FAST (Zhang et al., 2001). The Jacobian determinants are calculated from numerical integration and not analytical integration of functions used for nonlinear registration. The output of the Jacobian integration method is a percent change in volume; it is not a cross-sectional measure.

### 2.5 Effect of study designs

We evaluated the effect of image resolution and duration of trials using the Jacobian integration method. To assess the effect of image resolution, the rate of cortical atrophy was measured from higher-resolution sagittal MRIs (1.5 × 1.0 × 1.0 mm^3^) and separately using standard-resolution axial MRIs (1.0 × 1.0 × 3.0 mm^3^). It should be noted that this evaluation of image resolution does not test the pure effect of resolution change because the pulse timing parameters are not the same. Nonetheless, we find that this evaluation is more realistic than synthetic averaging of slices, and the result is directly applicable to real-world clinical trials.

For the statistical effect of the study duration, the rate of cortical atrophy was measured from baseline to year 1, baseline to year 2, and baseline to year 3.

### 2.6 Statistical analysis

For scan–rescan analysis, we measured the absolute percent volume change.

In order to determine and compare the statistical power of each pipeline, we estimated the sample size (per arm) required to detect pre-specified treatment effects (10–90%) in the longitudinal data set, without accounting for normal aging. We compared combinations of the following analysis pipelines (1) XPP + SPM, (2) LPP + SPM, (3) XPP + SIENAX, (4) LPP + SIENAX, (5) cross-sectional version of FreeSurfer, (6) longitudinal version of FreeSurfer, (7) XPP + Jacobian integration method, and (8) LPP + Jacobian integration method. The effect of study design was investigated using Jacobian integration method only.

We used the “pwr” package (http://cran.r-project.org/web/packages/pwr/index.html) in R to estimate the sample size required to detect treatment effects with 80% power, 0.05-significance level, and 10–90% treatment effects. The treatment effect was assumed to start immediately and remain constant over 3 years. The 95% confidence interval was estimated by bootstrapping 10,000 times. The sample size was calculated from the longitudinal data and independent of scan–rescan data.

For the analyses using SIENAX, SPM, and FreeSurfer, the atrophy rate was calculated from the difference in cortical gray matter volume, total gray matter volume, and cortical thickness, respectively. For the Jacobian integration method, the output is a direct measure of atrophy rate in percent change. The atrophy rates were annualized before calculating the sample sizes. As in Healy et al. (2009), we defined outliers a priori as having an annualized change greater than 10%/year, and these were eliminated from the analysis. The number of outliers for each technique is reported in Results section.

## 3 Results

The scan–rescan absolute percent differences in gray matter volume or thickness were 0.32 ± 0.23% (range = 0.01–0.85%) for the XPP + Jacobian integration method, 0.32 ± 0.24% (range = 0.03–0.90%) for the LPP + Jacobian integration method, 0.80 ± 0.73% (range = 0.05–2.85%) for XPP + SPM8, 0.89 ± 0.70% (range = 0.07–2.86%) for LPP + SPM8, 1.50 ± 1.41% (range = 0.01–6.41%) for XPP + SIENAX, 0.73 ± 0.57% (range = 0.01–2.57%) for LPP + SIENAX, and 1.04 ± 0.41% (range = 0.30–1.84%) for longitudinal FreeSurfer.

From the longitudinal dataset, we removed data from seven sites that changed scanners during the data acquisition period. We also did not analyze images with incomplete supratentorial brain coverage or severe artifacts. For high-resolution MRIs, there were 279 baseline and year-one image pairs, 159 baseline and year-two image pairs, and 71 baseline and year-three image pairs. The respective numbers were 274, 158, and 71 for the standard-resolution MRI image-pairs. There was no effect of treatment on whole brain or cortical atrophy, and the following analysis was performed on the combined group.

### 3.1 Evaluation of pipelines

**Fig. 2:**
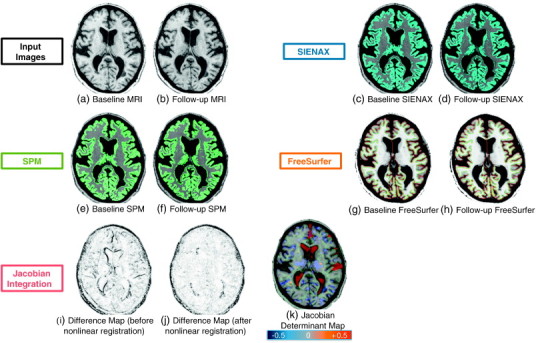
Example images from a single RRMS subject: (a) baseline high-resolution T1-weighted MRI, (b) linearly registered follow-up high-resolution T1-weighted MRI, (c) output of SIENAX for baseline MRI with colored gray matter tissue, (d) SIENAX from the follow-up scan, outputs of SPM on baseline MRI (e) and follow-up MRI (f). The surface result of FreeSurfer on baseline MRI (g) and on follow-up MRI (h). Finally, from the Jacobian integration method, the absolute intensity difference maps, (i) = before and (j) = after nonlinear registration, and (k) color-coded Jacobian determinant map where red indicates voxel expansion and blue indicates contraction (range = ± 50%). A marked enlargement is visible in the ventricles and frontal sulcus (red) while overall parenchymal atrophy is visible in cortical gray matter and thalami (blue).

**Fig. 3:**
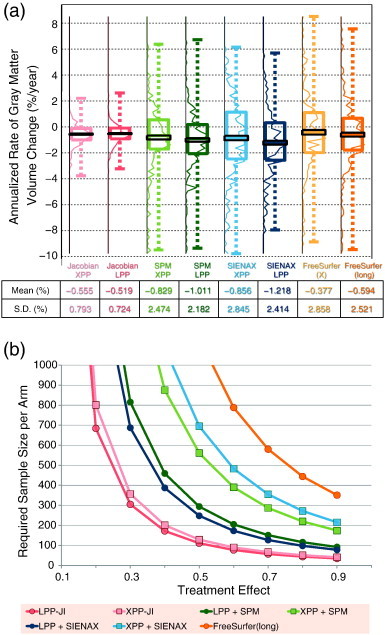
(a) Boxplot showing the percent change of cortical or total gray matter volume measured from each pipeline from one-year data after removing outliers. Jacobian integration and SIENAX measure cortical volume change while SPM measures total gray matter volume change. The colored boxplot shows the first quartile, median, third quartile, and extreme values; the black rectangle indicates the mean with standard error; the curves are the corresponding histograms. The darker curves use LPP, and lighter colors for XPP. Mean, standard deviation (SD), and effect size are shown below for each method. (b) The required sample size per arm for each pipeline for varying treatment effects with fixed power of 80% and 0.05 significance level. Table 1 shows the same values with 95% confidence interval. Values greater than 1000 are omitted here as such trials are not realistic. Cross-sectional FreeSurfer, FreeSurfer (x) values are not displayed because their values are above 1000. Abbreviations: XPP = cross-sectional pre-processing; LPP = longitudinal pre-processing.

Fig. 2 shows an example of input and resulting images from a single RRMS subject. Fig. 3(a) shows the percent volume change in cortical or total gray matter atrophy from each pipeline, and Fig. 3(b) shows the required sample size for varying treatment effects. Table 1 shows the corresponding values with 95% confidence intervals. The numbers of outliers (defined as > 10%/year change a priori) was none from Jacobian integration method; 4 from SPM8 and SIENAX with XPP; and 2 from SPM8 and SIENAX with LPP; 16 for cross-sectional FreeSurfer and 4 for longitudinal FreeSurfer. The mean annualized rates (SD) of atrophy were − 0.555 (0.793), − 0.519 (0.724), − 0.829 (2.474), − 1.011 (2.182), − 0.856 (2.845), − 1.218 (2.414), − 0.377 (2.858), and − 0.594 (2.521) %/year for Jacobian integration, XPP + Jacobian, LPP + Jacobian, XPP + SPM8, LPP + SPM8, XPP + SINEAX, LPP + SIENAX, cross-sectional FreeSurfer, and longitudinal FreeSurfer, respectively. Compared to XPP, LPP decreased the required sample size on average by 38% and 57% for SPM8 and SIENAX, respectively. The Jacobian integration method showed further improvement and had a 58% reduction on average compared to the next best result, LPP + SPM8. Compared to conventional XPP + SIENAX, Jacobian integration reduced the sample size required to see a change in cortical gray matter by more than 5 fold.

### 3.2 Effect of study designs

**Fig. 4:**
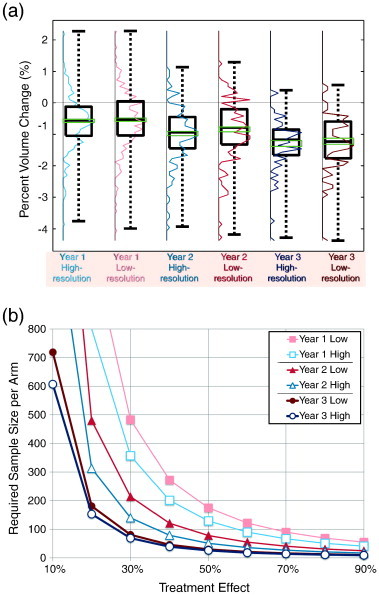
(a) Boxplots and histogram of cortical volume change comparing study durations and image resolutions using the Jacobian integration method. From left to right (year-one high-resolution, year-one low-resolution, year-two high-resolution, year-two low-resolution, year-three high-resolution, and year-three low-resolution), the average rates of cortical atrophy were − 0.56 ± 0.79, − 0.52 ± 0.87, − 0.48 ± 0.43, − 0.42 ± 0.47, − 0.43 ± 0.27, and − 0.41 ± 0.28% per year. (b) The sample size per arm required for varying treatment effect with fixed power of 80% and 0.05 significance level. The use of high resolution MRIs reduced the required sample size by 34% on average. The reduction was greater for short duration or small treatment effect. Longer study duration consistently decreased the required sample size (41% per year on average).

MRI image resolution was found to be a significant contributor to study power. As shown in Fig. 4 and Table 2, low-resolution MRIs (3 mm slice thickness) required an average of 34% more subjects to detect differences. The same figure also shows that longer studies require fewer patients. A post-hoc analysis with a subset of patients who had completed 4 MRIs did not significantly change these results.

## 4 Discussion

The results of the current study showed that the longitudinal Jacobian integration method was superior to commonly-used cross-sectional methods — reducing the required sample size by 4–5 fold in the MS population studied. The required sample size was reduced when commonly-used cross-sectional methods were applied on longitudinally-pre-processed images (Nakamura et al., 2012), but the improvement with the Jacobian integration method far exceeded that improvement. Our results suggest that longitudinal methods such as the Jacobian integration method have substantial advantages for measuring cortical and gray matter atrophy in future clinical trials.

The fact that the average atrophy rates varied from − 0.519%/year with the Jacobian integration method + LPP to − 1.218%/year with LPP + SIENAX emphasizes that the interpretation of atrophy data requires caution. We cannot directly compare atrophy rates across different analysis methods. The current literature on cortical atrophy using Jacobian integration methods in MS is limited. In Anderson et al. (2009), the authors used a Jacobian integration method to compare the rates of gray matter atrophy in normal controls versus that in patients with RRMS and failed to detect a significant difference — likely due to the study's small sample size. In another study, Anderson et al. applied a Jacobian integration method in patients with Alzheimer's disease and showed a pattern similar to our findings of power improvement with respect to SIENAX (Anderson et al., 2012).

The estimated sample size required by SIENAX in the current study was larger than that of a previous report by Healy et al. (2009). The latter study reported approximately 70–250 patients per arm for a 2-year annual MRI study with 50% treatment effect and 80% power, whereas our equivalent analysis with XPP–SIENAX required approximately 450 patients per arm (data not shown). A number of important differences between the studies may underlie this discrepancy: first, study designs were different (measurement of *total* gray matter atrophy from *monthly* MRIs in Healy et al. vs. *cortical* atrophy from *annual* MRI in the current study); and, second, the atrophy rates in the patient populations were very different (from − 1.9 to − 3.6%/year in Healy et al. and from − 0.56% to − 1.22% in the current study). It is plausible that the MS population studied here, all of whom were on treatment with glatiramer acetate, was more stable than the population in that study.

The current study did not include a placebo arm, which would have allowed for an estimation of actual treatment effect. Nevertheless, future MS clinical trials are unlikely to have placebo arms due to ethical considerations (Polman et al., 2008); therefore, modeling the treatment effect against a placebo population may overestimate the statistical power when applied to a trial with an active comparator arm. As a result, our estimations are more directly applicable to future clinical trials. The current study also did not examine cortical atrophy in normal controls, which could have allowed us to account for normal aging. However, cortical, or gray matter, atrophy due to normal aging is very small compared with MS-related atrophy at the group level (− 0.028 ± 0.24 and − 0.23 ± 0.34% per year, respectively, for normal controls and patients with RRMS in Fisher et al. (2008); and − 0.06 ± 0.16 and − 0.15 ± 0.27% per year in Anderson et al. (2009)); thus, we believe that neglecting normal aging effects in the present calculation introduces little error. However, in a population where atrophy due to normal aging is not small (e.g., Alzheimer's disease), this approach could overestimate the power.

FreeSurfer did not perform well in the current study as shown by the large sample sizes and many outliers. It is possible that images used in the longitudinal study (single FLASH without signal averaging or multi-echo) are not optimal for FreeSurfer. Nonetheless, it emphasizes that the Jacobian integration method is robust with respect to the T1-weighted sequence details as its performance in low-resolution images is relatively similar to that from high-resolution MRIs. This is a potentially important practical advantage of Jacobian integration, as it can be used almost equally as well on images with 3 mm slice thickness, which is standardly acquired in MS clinical trials.

We measured atrophy in all cerebral gray matter (deep and cortical gray matter) directly using different methods, and compared their statistical power. Cortical gray matter forms about 80% of total gray matter and does not include deep structures such as the thalami, which are known to show disproportionately high rates of atrophy, even in early MS (Henry et al., 2008). Importantly, cortical gray matter also does not include cerebellar gray matter, and the segmentation of which on conventional MRI scans is highly contaminated by partial volume effects. Sample sizes were not very different for the two cross-sectional approaches (namely, SPM and SIENAX), and all things considered, it is difficult to assess whether one metric should be preferred in the clinical trial setting.

A constant and immediate treatment effect was assumed here. However, it is possible that gray matter may demonstrate pseudoatrophy (an acceleration of atrophy in the first year following the initiation of treatment), depending on the nature of the treatment being initiated (Zivadinov et al., 2008). Previous studies have suggested that white matter volume change is predominantly affected by pseudoatrophy whereas gray matter is less sensitive to fluctuations associated with changes in inflammation (Nakamura et al., 2010; Tiberio et al., 2005). However, we cannot exclude the possibility that pseudoatrophy can occur in gray matter as well (Horakova et al., 2008; Nakamura et al., 2010). It is also possible that there is a tissue-specific delayed effect of treatment on atrophy; that is, conventional anti-inflammatory treatments may reduce inflammation in white matter first, followed by reduced Wallerian degeneration, and ultimately neuroprotection. Such treatment-specific mechanisms of action may also play a role in the dynamics of cortical atrophy.

In conclusion, our results clearly show that longitudinal registration-based methods such as the Jacobian integration method described here have greater statistical power for detecting treatment effects on gray matter atrophy than the commonly-used cross-sectional segmentation-based methods, even when the latter are combined with longitudinal pre-processing. Our results should help in the planning of new clinical trials assessing neuroprotection in MS.

## Figures and Tables

**Table 1:** Comparison of various pipelines.

Treatment effect	XPP + Jacobian integration	LPP + Jacobian integration	XPP + SPM	LPP + SPM	XPP + SIENAX	LPP + SIENAX	Cross-sectional FreeSurfer	Longitudinal FreeSurfer
10%	3201 (2129–4972)	2731 (1996–3888)	> 10,000 (7178–)	7321 (4178–)	> 10,000 (8143–)	6174 (3638–)	> 10,000	> 10,000
20%	801 (533–1244)	684 (500–973)	3496 (1796–9657)	1832 (1045–3756)	4336 (2037–)	1545 (911–2990)	> 10,000 (6339–)	7084 (3184–)
30%	357 (238–554)	305 (223–433)	1,555 (799–4293)	815 (465–1670)	1928 (906–6764)	687 (406–1330)	> 10,000 (2818–)	3148 (1416–)
40%	201 (134–312)	172 (126–244)	875 (450–2415)	459 (262–940)	1085 (510–3805)	387 (229–748)	5645 (1586–)	1772 (797–6137)
50%	129 (87–200)	111 (81–157)	561 (289–1546)	294 (169–602)	695 (327–2436)	248 (147–480)	3613 (1016–)	1134 (511–3928)
60%	90 (61–140)	77 (57–109)	390 (201–1074)	205 (117–419)	483 (228–1692)	173 (102–333)	2510 (706–)	788 (355–2728)
70%	67 (45–103)	57 (42–81)	287 (148–790)	151 (87–308)	355 (168–1244)	127 (76–245)	1844 (519–)	580 (261–2005)
80%	51 (35–79)	44 (33–62)	220 (114–605)	116 (67–236)	272 (129–952)	98 (58–188)	1412 (398–)	444 (200–1535)
90%	41 (28–63)	35 (26–49)	174 (90–478)	92 (53–187)	215 (102–753)	78 (46–149)	1116 (314–)	351 (159–1213)

Required sample size per arm to detect treatment effect on cortical atrophy except for SPM, which uses gray matter atrophy. The range is a 95% confidence interval obtained from bootstrapping 10,000 times. Abbreviations: XPP = cross-sectional pre-processing; LPP = longitudinal pre-processing.

**Table 2:** Effect of resolution and duration on the required sample size per arm to detect treatment effect on cortical atrophy.

Treatment effect	Year 1 high-resolution	Year 1 low-resolution	Year 2 high-resolution	Year 2 low-resolution	Year 3 high-resolution	Year 3 low-resolution
10%	3201 (2269–4609)	4331 (2277–4586)	1247 (899–1717)	1917 (1372–2707)	607 (367–885)	719 (437–1076)
20%	801 (568–1153)	1084 (570–1148)	313 (226–430)	480 (344–678)	153 (93–222)	181 (110–270)
30%	357 (253–513)	483 (254–511)	140 (101–192)	214 (154–302)	69 (42–100)	81 (50–121)
40%	201 (143–289)	272 (144–288)	79 (58–109)	121 (87–171)	39 (24–57)	46 (29–69)
50%	129 (92–186)	175 (93–185)	51 (37–70)	78 (56–110)	26 (16–37)	30 (19–44)
60%	90 (64–129)	122 (65–129)	36 (26–49)	55 (40–77)	18 (12–26)	21 (14–31)
70%	67 (48–96)	90 (48–95)	27 (20–36)	41 (29–57)	14 (9–20)	16 (10–23)
80%	51 (37–73)	69 (37–73)	21 (16–28)	31 (23–44)	11 (7–15)	13 (8–18)
90%	41 (29–58)	55 (30–58)	17 (13–23)	25 (18–35)	9 (6–12)	10 (7–15)

Required sample size per arm to detect treatment effect on cortical atrophy using the Jacobian integration method. The range is a 95% confidence interval obtained from bootstrapping 10,000 times.
